# P-750. Doxycycline is Inferior to Cephalexin and Sulfamethoxazole/Trimethoprim (SMX/TMP) for the Treatment of Culture Negative Acute Bacterial Skin and Skin Structure Infections (ABSSSIs)

**DOI:** 10.1093/ofid/ofaf695.961

**Published:** 2026-01-11

**Authors:** Jeffrey W Jansen, Michael Lorenz, Ryan P Moenster, Travis W Linneman, Ashleigh Wallace-Lacey

**Affiliations:** VA Midsouth Healthcare Network Clinical Resource Hub, St. Peter's, MO; VA St. Louis HCS, St. Louis, Missouri; St. Louis College of Pharmacy at UHSP/VA St. Louis Health Care System, St. Louis, Missouri; VA St. Louis HCS, St. Louis, Missouri; VA St. Louis HCS, St. Louis, Missouri

## Abstract

**Background:**

The 2014 IDSA guidelines recommend empiric treatment for ABSSSI based on categorization of purulent or non-purulent as this provides insight to the causative pathogen. However, in clinical practice, methicillin-resistant *Staphylococcus aureus* (MRSA) therapy is commonly prescribed in non-purulent infections or infections where serous drainage is mistaken for purulence. Doxycycline is often prescribed for ABSSSI despite limited and antiquated data for its use as a monotherapy option. The current analysis aims to compare the clinical failure rates in patients treated for culture negative ABSSSI with either doxycycline, cephalexin, or SMX/TMP.

**Table 1:**
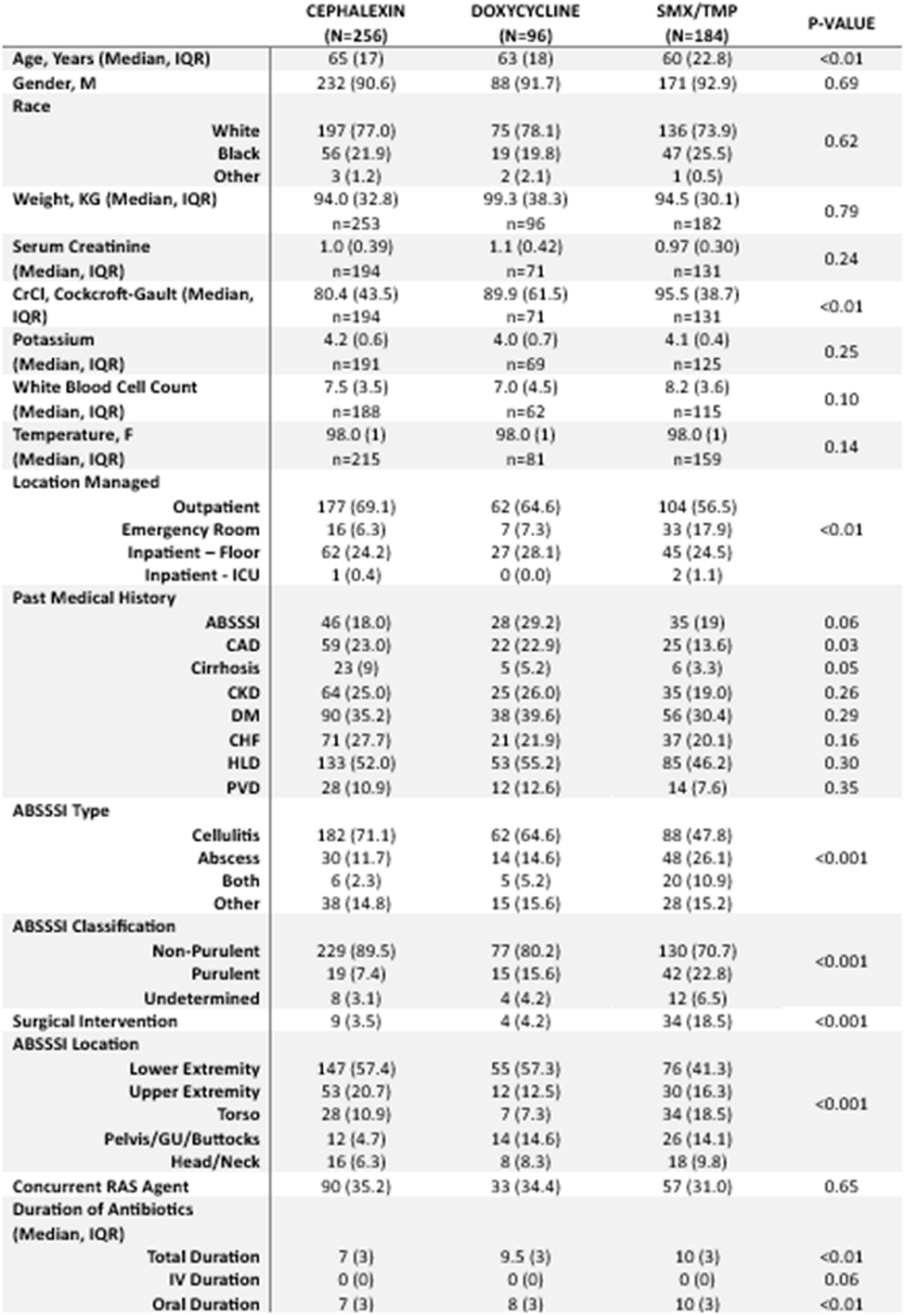
Baseline Characteristics

**Table 2:**
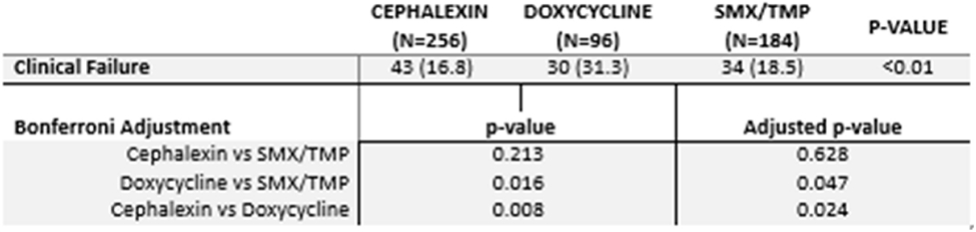
Clinical Failure vs Antibiotic Received Adjusted p-value using the Bonferroni Correction

**Methods:**

A retrospective cohort was completed from patients at the VA St Louis Healthcare System aged 18 years through 89 years of age treated for an ABSSSI from either a VA clinic, the emergency room (ER), or inpatient with doxycycline, cephalexin, or TMP/SMX therapy between 10/01/2015 and 12/31/2022. The primary outcome was a composite of clinical failure defined as meeting any of the following within 28 days of antibiotic end date: (1) representation for further evaluation of infection at the same anatomical site; (2) extension or change in antimicrobial agent; and (3) unplanned surgical procedure for treatment of index infection. Key exclusion criteria were patients who received > 72 hours intravenous antimicrobial therapy, combination therapy, positive cultures that influenced antibiotic decision, and deep-seated infections.

**Table 3:**
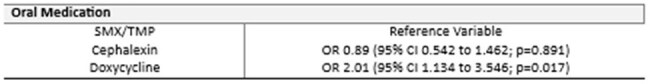
Unadjusted Oral Medication vs Clinical Failure

**Table 4:**
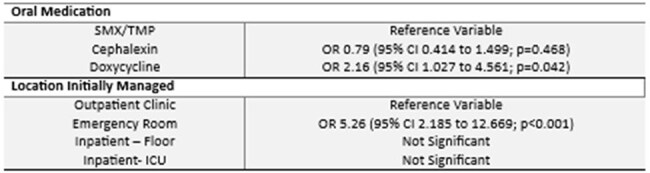
Adjusted* Oral Medication vs Clinical Failure *Model controlled for age, creatinine clearance, total antibiotic duration, white blood cell count, history of acute bacterial skin and skin structure infection (ABSSSI), history of coronary artery disease, history of cirrhosis, surgical intervention completed at presentation, ABSSSI type, ABSSSI classification, ABSSSI anatomic location, and location managed.

**Results:**

Overall, 536 patients were included with baseline characteristics are provided in table 1. Thirty patients (31.3%) in the doxycycline group experienced clinical failure, which was statistically significant when compared to cephalexin (43, 16.8%; p = 0.024) and SMX/TMP (34, 18.5%; p = 0.047). Failure rates between SMX/TMP and cephalexin were not significantly different (p = 0.628) (Table 2). Doxycycline therapy was associated with increased clinical failure rates in the regression analysis (Tables 3 and 4).

**Conclusion:**

Doxycycline is inferior to therapy with cephalexin or SMX/TMP for culture negative ABSSSI regardless of infection classification, location, or type.

**Disclosures:**

All Authors: No reported disclosures

